# Early endocrine diagnoses and subsequent survival after immune checkpoint inhibitor initiation in non-small cell lung cancer: a 180-day landmark cohort study

**DOI:** 10.3389/fonc.2026.1957383

**Published:** 2026-09-17

**Authors:** Shih-Sen Lin, Pei-Sung Hsu, Hung-Chi Lai, Ju-Fang Liu, Wen-Pin Cheng

**Affiliations:** 1 Graduate Institute of Clinical Medicine, College of Medicine, National Taiwan University, Taipei, Taiwan; 2 Division of Chest Medicine, Department of Internal Medicine, Shin Kong Wu Ho-Su Memorial Hospital, Taipei, Taiwan; 3 Clinical Trial Center, Department of Research, Shin Kong Wu Ho-Su Memorial Hospital, Taipei, Taiwan; 4 Division of Hematology and Oncology, Department of Internal Medicine, Shin Kong Wu Ho-Su Memorial Hospital, Taipei, Taiwan; 5 Translational Medicine Center, Department of Research, Shin Kong Wu Ho-Su Memorial Hospital, Taipei, Taiwan; 6 School of Oral Hygiene, College of Oral Medicine, Taipei Medical University, Taipei, Taiwan; 7 Graduate Institute of Human Resource and Knowledge Management, National Kaohsiung Normal University, Kaohsiung, Taiwan

**Keywords:** carcinoma, endocrine system diseases, immune checkpoint inhibitors, non-small cell lung cancer, prognosis, survival analysis

## Abstract

**Background:**

Endocrine diagnoses newly recorded after immune checkpoint inhibitor (ICI) initiation may be associated with subsequent outcomes, but time-dependent bias and the limitations of diagnosis-code attribution complicate interpretation.

**Methods:**

We conducted a retrospective 180-day landmark cohort study using a federated electronic health record network. Patients with non–small-cell lung cancer (NSCLC) initiating ICI were classified according to the presence or absence of a newly recorded qualifying thyroid, pituitary, or adrenal diagnosis during days 1–180 after first ICI. Both cohorts excluded qualifying endocrine diagnoses during days −365 to −1 and required a recorded visit during months 5–6. After 1:1 propensity-score matching, 7,696 patients were included in each group. Outcomes were assessed from day 181.

**Results:**

Newly recorded endocrine diagnoses were associated with lower subsequent mortality (hazard ratio [HR], 0.782; 95% confidence interval [CI], 0.745–0.821; P < 0.0001) and a lower hazard of first recorded prespecified subsequent systemic therapy or death in an independently matched analysis (HR, 0.849; 95% CI, 0.773–0.934; P = 0.0007). Exploratory severe infection (HR, 1.255; 95% CI, 1.151–1.369) and healthcare utilization (HR, 1.055; 95% CI, 1.013–1.099) outcomes occurred more frequently in the exposed group. Findings were directionally consistent across subgroup and sensitivity analyses, including a repeat-code definition.

**Conclusions:**

Among patients satisfying the 180-day landmark and observability criteria, newly recorded endocrine diagnoses after ICI initiation were associated with lower subsequent mortality. This association is conditional on the specified landmark and observability criteria and does not establish ICI attribution, causality, or prediction of comparative treatment benefit.

## Introduction

Immune checkpoint inhibitors (ICIs) targeting the programmed cell death-1 (PD-1)/programmed death-ligand 1 (PD-L1) pathway have transformed treatment for non–small-cell lung cancer (NSCLC), improving overall survival (OS) as monotherapy, in combination with platinum-based chemotherapy, and as consolidation after definitive chemoradiotherapy in selected settings (1–3). Nevertheless, clinical outcomes vary substantially, and available baseline biomarkers do not fully identify patients who will achieve durable disease control. Clinically attributed immune-related adverse events (irAEs) have been associated with treatment response and survival, although the strength and direction of these associations vary by organ system, event severity, and study design (4). Endocrine irAEs are of particular interest because they are relatively frequent, are often manageable with hormone replacement, and may be recorded in routine electronic health records. However, observational analyses of post-treatment events are vulnerable to guarantee-time (immortal-time) bias because patients must remain alive and observed long enough to receive a diagnosis (5). In addition, a structured diagnosis code alone cannot establish clinician attribution, severity, or a causal relationship to ICI therapy. We therefore evaluated the association between newly recorded thyroid, pituitary, or adrenal diagnoses during days 1–180 after first ICI and subsequent mortality among patients with NSCLC satisfying a 180-day landmark and observability criterion. We used harmonized baseline eligibility, propensity-score matching, subgroup analyses, and prespecified sensitivity analyses while restricting interpretation to a conditional association.

## Materials and methods

### Data source and design

The present study was a retrospective 180-day landmark cohort study using data from the TriNetX LIVE Global Collaborative Network (TriNetX, LLC, Cambridge, MA, USA), a federated network of electronic health records from participating healthcare organizations. The network captures routinely recorded demographic characteristics, diagnoses, procedures, medications, and encounters. Data were accessed on August 16, 2026; 180 healthcare organizations responded to the locked primary query. All analyses were performed within the platform, and only deidentified aggregate results were available.

### Ethics, institutional review board, and informed consent

The Review Committee at Shin Kong Wu Ho-Su Memorial Hospital approved this retrospective study and waived the requirement for informed consent (approval No. 20260301R). The study analyzed previously collected data and did not involve direct contact with patients or any requirement for patient participation. Information accessed through the TriNetX platform was deidentified in accordance with the deidentification criteria outlined in Section §164.514(a) of the Health Insurance Portability and Accountability Act Privacy Rule. The deidentification procedure was validated through a formal assessment by a qualified expert, as specified in §164.514(b) (1).

### Study population and index date

Patients were eligible if a diagnosis of NSCLC was recorded 1 day to 5 years before the first recorded administration of a prespecified PD-1, PD-L1, or cytotoxic T-lymphocyte–associated protein 4 (CTLA-4) inhibitor. Records indicating small-cell carcinoma were excluded. The first recorded ICI administration in the available data was the index date. Eligible ICI index dates occurred from January 1, 2010, through January 31, 2026. The NSCLC criterion served only as an eligibility relation and did not define the index event.

Endocrine diagnosis definition and landmark framework.

Early endocrine diagnosis status was defined by at least one newly recorded qualifying diagnosis-group instance for thyroid dysfunction or thyroiditis, pituitary dysfunction, or adrenal disease during days 1–180 after the index date. The control cohort had no qualifying instance during this window. Both cohorts excluded qualifying endocrine diagnoses recorded during days −365 to −1. Full concepts and temporal rules are provided in **Supplementary Table S1**. Because continuous enrollment could not be established in the federated EHR data, both cohorts were required to have at least one recorded visit during months 5–6 after first ICI. This criterion was applied identically during cohort construction and before matching as an operational proxy for ongoing contact with the network near the 180-day landmark; it did not establish uninterrupted enrollment or complete follow-up. Exposure ascertainment ended on day 180, and outcome follow-up began on day 181.

## Outcomes

The primary outcome was overall mortality, identified by the platform deceased status and prespecified mortality-related diagnosis codes. After propensity-score matching, the platform option to exclude patients with the outcome before the analysis window was applied identically to both cohorts. For the OS window beginning on day 181, patients with a mortality outcome recorded before day 181 were removed before Kaplan–Meier and Cox estimation. This outcome-specific postmatching risk-set restriction was not an additional prematching eligibility criterion. Secondary outcomes were time to first recorded prespecified subsequent systemic therapy or death (TTNT-D), severe infection, and healthcare utilization. TTNT-D was analyzed in an independent outcome-specific cohort pair derived from the primary definitions: patients with any qualifying subsequent-therapy code from the beginning of available records through day 180 were excluded before matching, the remaining cohorts were independently matched 1:1, and no additional outcome-level prior-event exclusion was applied. Severe infection and healthcare utilization were analyzed in the primary matched cohort without excluding prior recurrent events. Multi-component endpoints used Boolean OR logic, and all outcomes were assessed during days 181–1825. Full definitions, component code lists, risk-set rules, and event counts are provided in **Supplementary Table S4**. Because formal competing-risk regression was unavailable, severe infection and healthcare utilization were considered exploratory nonmortality outcomes. The platform-generated Cox models did not formally model death as a competing event; the reported HRs therefore should not be interpreted as cumulative-incidence estimates or Fine–Gray subdistribution HRs.

### Covariates, matching, and statistical analysis

All diagnosis-, procedure-, and medication-based propensity-score covariates were assessed during days −365 to −1. Age was measured at index, and sex and race were obtained from demographic records. The model included age; sex; White, Black or African American, and Asian race; liver, bone, and brain metastases; diabetes mellitus; chronic obstructive pulmonary disease; chronic kidney disease; heart failure; nicotine dependence; long-term systemic steroid use; prior antineoplastic radiation therapy; prior antineoplastic agents; and immunosuppressants. No post-index variable was included, and concomitant chemotherapy was retained only as a subgroup variable. The platform estimated propensity scores and performed 1:1 matching using its built-in algorithm. Balance was assessed using absolute standardized mean differences (SMDs), with <0.10 prespecified as acceptable. The complete model and balance are reported in **Supplementary Table S2**; a Love plot and platform-generated propensity-score distributions are shown in **Supplementary Figures S1**, **S2**. Kaplan–Meier methods, log-rank tests, and platform Cox models were used to estimate HRs and 95% CIs. Patients were censored after the last recorded fact in the platform. The Cox models were estimated by the platform within the 1:1 propensity-score-matched cohorts; however, the aggregate output did not document matched-pair stratification or cluster-robust variance estimation. Because patient-level matched-pair identifiers were unavailable for export, within-pair dependence could not be independently incorporated into variance estimation.

### analyse Subgroups and sensitivity s

Prespecified subgroup analyses were performed for sex, age (<65, 65–74, and ≥75 years), mutually exclusive ICI class, metastatic disease, baseline systemic steroid use, and concomitant chemotherapy. PD-1-only and PD-L1-only cohorts excluded the other ICI classes within 1 month on or after first ICI, whereas the CTLA-4-containing cohort permitted concomitant PD-1/PD-L1 exposure. Baseline steroid status used the period from 3 months and 1 day before first ICI through the day before index, and concomitant chemotherapy used the interval from 1 month before to 1 month after first ICI. Each subgroup cohort was independently reconstructed and independently matched; its denominator therefore represented a distinct analytic population and was not expected to sum to the primary matched cohort. Between-stratum P values for heterogeneity were calculated from subgroup-specific log HRs and standard errors derived from 95% CIs using a summary-level inverse-variance test, not a patient-level Cox interaction model. The subgroup audit is provided in **Supplementary Table S3**. Sensitivity analyses used 90- and 120-day landmarks with outcome follow-up beginning on days 91 and 121, respectively; compared diagnoses during days 1–90 or 91–180 with no diagnosis through day 180; and required at least two qualifying diagnosis-group instances during days 1–180. The LIVE interface could not confirm that repeat-code instances occurred on separate dates.

## Results

The complete Boolean and temporal definitions jointly identified 8,531 patients with an early endocrine diagnosis and 24,323 without one (**Figure 1**). After applying propensity-score eligibility, 7,696 and 22,273 patients, respectively, were available before matching; 1:1 matching yielded 7,696 patients in each cohort. Query N denotes the number satisfying the complete joint definition; mutually exclusive criterion-by-criterion attrition was not available from the aggregate interface and was not reconstructed. **Table 1** presents baseline characteristics before and after matching. All postmatching absolute SMDs were <0.10, with improved balance also shown in **Supplementary Figures S1**, **S2**.

**Figure 1:**
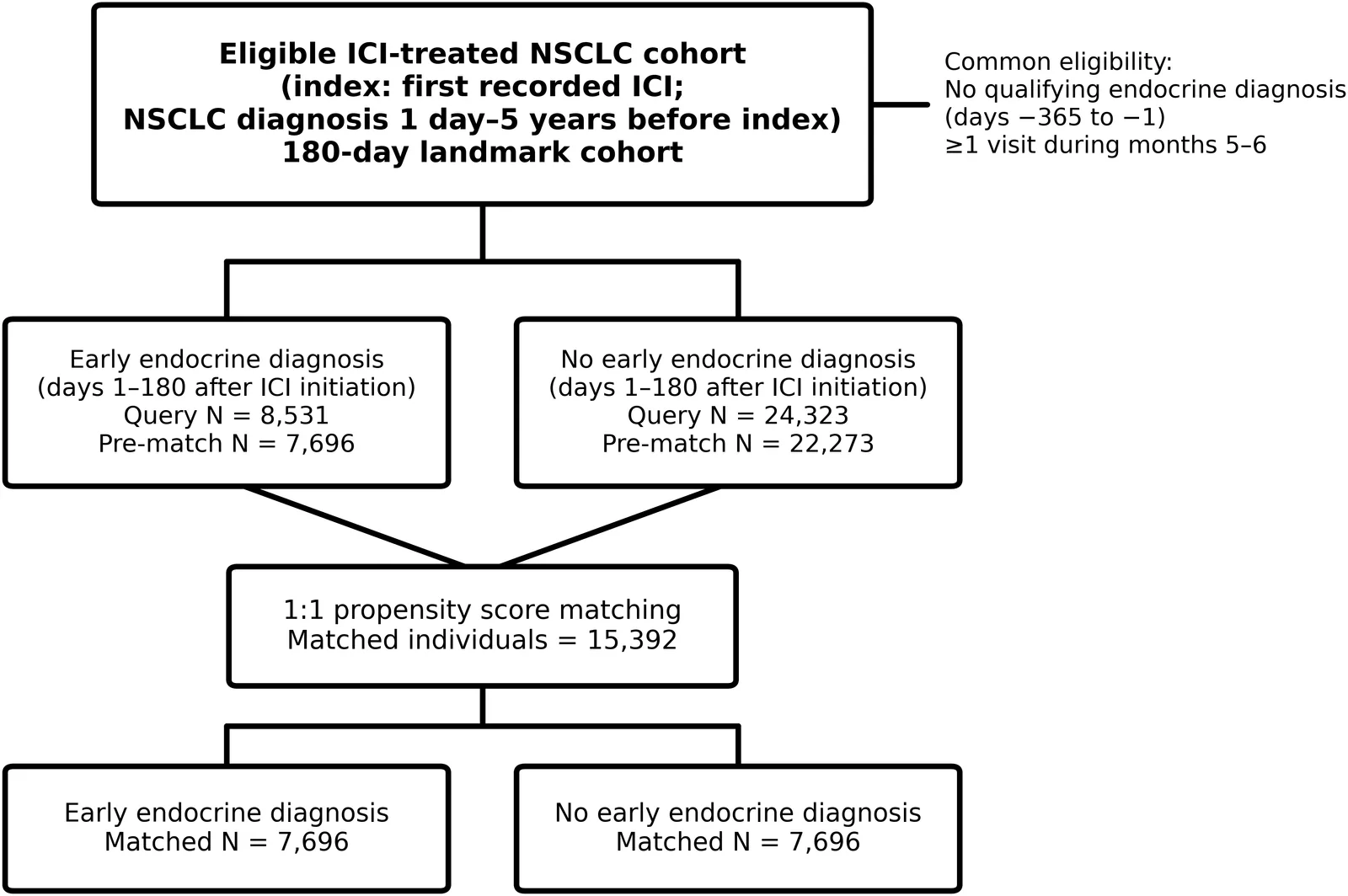
Study flow diagram. Study flow diagram illustrating cohort assembly. Eligible patients had NSCLC recorded 1 day to 5 years before the first recorded ICI administration, no qualifying endocrine diagnosis during days −365 to −1, and at least one recorded visit during months 5–6 as an operational proxy for observability near the landmark. Exposure was classified during days 1–180 after first ICI. Query N denotes the number satisfying the complete Boolean and temporal definition jointly; mutually exclusive criterion-specific attrition was not available from the aggregate interface and was not reconstructed. Complete-query N was 8,531 versus 24,323, the propensity-score-matching-eligible prematch N was 7,696 versus 22,273, and 1:1 matching yielded 7,696 patients per cohort.

**Table 1:** Baseline characteristics of cohorts before and after propensity score matching.

	Before matching			After matching		
Clinical characteristics	Early endocrine diagnosis (n=7,696)	No early endocrine diagnosis (n=22,273)	SMD	Early endocrine diagnosis (n=7,696)	No early endocrine diagnosis (n=7,696)	SMD
Age at index						
Mean± SD	66.6 +/- 10.2	66.8 +/- 10.4	0.015	66.6 +/- 10.2	66.6 +/- 10.1	0.004
Gender						
Female	3,641 (47.3%)	9,400 (42.2%)	0.103	3,641 (47.3%)	3,620 (47.0%)	0.005
Male	4,055 (52.7%)	12,871 (57.8%)	0.103	4,055 (52.7%)	4,075 (52.9%)	0.005
Race						
White	5,903 (76.7%)	14,379 (64.6%)	0.269	5,903 (76.7%)	5,909 (76.8%)	0.002
Black or African American	777 (10.1%)	2,330 (10.5%)	0.012	777 (10.1%)	777 (10.1%)	<0.001
Asian	335 (4.4%)	1,466 (6.6%)	0.098	335 (4.4%)	356 (4.6%)	0.013
Comorbidities						
Other chronic obstructive pulmonary disease	2,272 (29.5%)	6,058 (27.2%)	0.052	2,272 (29.5%)	2,251 (29.2%)	0.006
Diabetes mellitus	1,418 (18.4%)	4,006 (18.0%)	0.011	1,418 (18.4%)	1,357 (17.6%)	0.021
Chronic kidney disease (CKD)	682 (8.9%)	2,036 (9.1%)	0.010	682 (8.9%)	595 (7.7%)	0.041
Heart failure	579 (7.5%)	1,671 (7.5%)	0.001	579 (7.5%)	530 (6.9%)	0.025
Metastatic conditions						
Secondary malignant neoplasm of brain	1,361 (17.7%)	3,248 (14.6%)	0.084	1,361 (17.7%)	1,308 (17.0%)	0.018
Secondary malignant neoplasm of liver and intrahepatic bile duct	625 (8.1%)	1,741 (7.8%)	0.011	625 (8.1%)	604 (7.8%)	0.010
Secondary malignant neoplasm of bone	1,459 (19.0%)	3,862 (17.3%)	0.042	1,459 (19.0%)	1,446 (18.8%)	0.004
Smoking and treatment-related variables						
Nicotine dependence	2,431 (31.6%)	6,010 (27.0%)	0.101	2,431 (31.6%)	2,470 (32.1%)	0.011
Long term (current) use of systemic steroids	379 (4.9%)	940 (4.2%)	0.034	379 (4.9%)	323 (4.2%)	0.035
Encounter for antineoplastic radiation therapy	769 (10.0%)	1,627 (7.3%)	0.096	769 (10.0%)	708 (9.2%)	0.027
Antineoplastic agents	2,698 (35.1%)	8,086 (36.3%)	0.026	2,698 (35.1%)	2,580 (33.5%)	0.032
Immunosuppressants	122 (1.6%)	332 (1.5%)	0.008	122 (1.6%)	97 (1.3%)	0.027

For OS, after matching, the same platform prior-outcome rule was applied to both cohorts before Kaplan–Meier and Cox estimation. It excluded 560 patients with an early endocrine diagnosis and 762 without one who had mortality recorded before day 181, producing analytic risk sets of 7,136 and 6,934 patients, with 3,185 and 3,312 deaths, respectively. From day 181, newly recorded endocrine diagnoses were associated with lower subsequent mortality (HR, 0.782; 95% CI, 0.745–0.821; log-rank P < 0.0001) (**Figure 2**).

**Figure 2:**
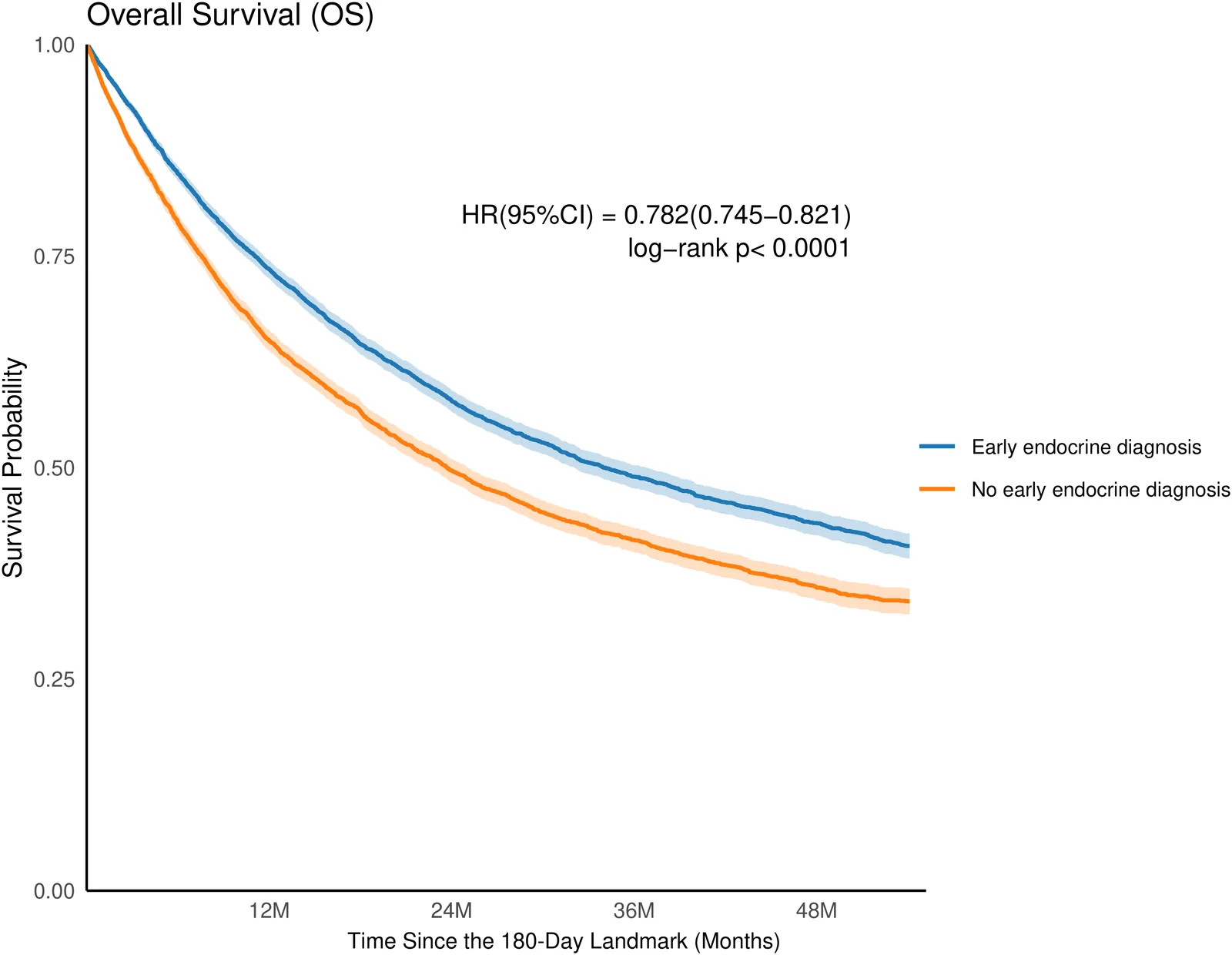
Kaplan–Meier overall survival. Kaplan–Meier curves for OS in the propensity-score-matched cohorts using a 180-day landmark design. Exposure was classified as an early endocrine diagnosis versus no early endocrine diagnosis during days 1–180 after first ICI, and survival follow-up began on day 181. After matching, the same platform prior-outcome exclusion rule was applied to both cohorts before survival estimation, producing OS analytic risk sets of 7,136 and 6,934 patients, respectively. Time on the horizontal axis is measured from the 180-day landmark. The HR and log-rank P value represent post-landmark comparisons. HR, hazard ratio; ICI, immune checkpoint inhibitor; OS, overall survival.

In the independently matched TTNT-D analysis, each cohort included 1,876 patients; events occurred in 866 exposed and 843 control patients, and the hazard was lower in the early endocrine diagnosis cohort (HR, 0.849; 95% CI, 0.773–0.934; P = 0.0007) (**Figure 3**). Exploratory severe infection occurred in 1,265/7,696 versus 866/7,696 patients (HR, 1.255; 95% CI, 1.151–1.369; P < 0.0001), and healthcare utilization occurred in 4,984/7,696 versus 4,306/7,696 patients (HR, 1.055; 95% CI, 1.013–1.099; P = 0.0101). These Cox HRs for severe infection and healthcare utilization are not cumulative-incidence estimates or Fine–Gray subdistribution HRs.

**Figure 3:**
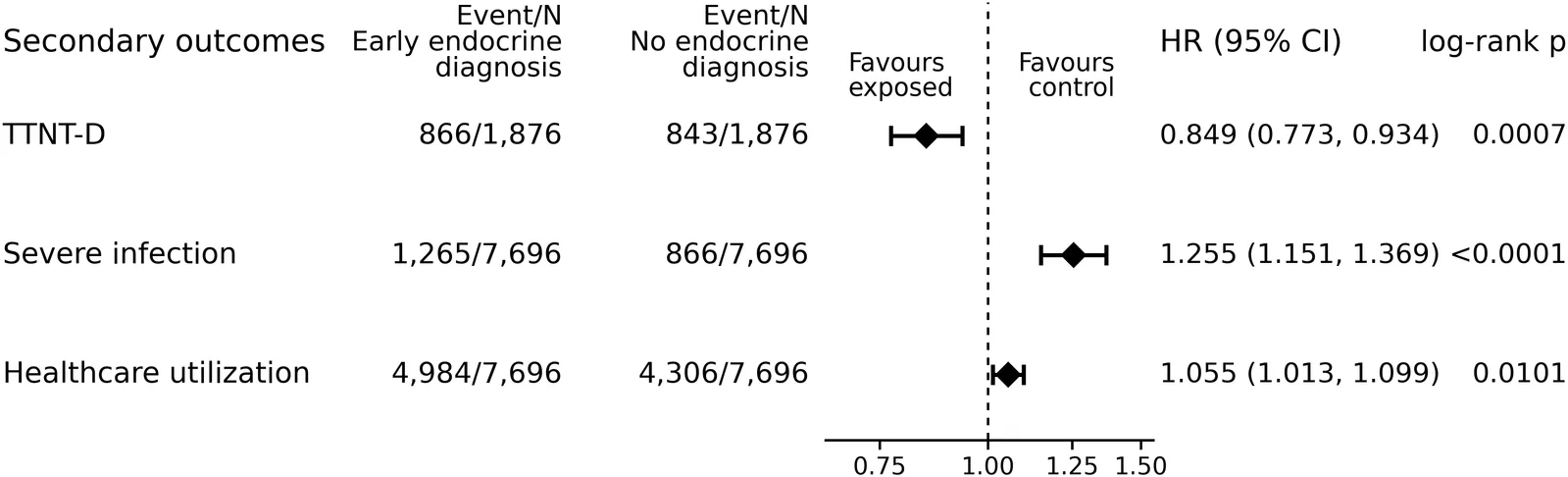
Secondary outcomes forest plot. Forest plot of revised secondary outcomes. TTNT-D was analyzed in an independently constructed and matched outcome-specific cohort after exclusion of qualifying subsequent-therapy codes through day 180; severe infection and healthcare utilization were analyzed in the primary matched cohort. Point estimates are HRs with 95% CIs, and event counts with analytic denominators are displayed. TTNT-D was defined as the first recorded prespecified subsequent systemic therapy or death. Severe infection and healthcare utilization were exploratory nonmortality outcomes because formal competing-risk regression was unavailable. These HRs are not cumulative-incidence or Fine–Gray subdistribution estimates. Values <1.0 indicate a lower hazard in the early endocrine diagnosis cohort, whereas values >1.0 indicate a higher hazard. CI, confidence interval; HR, hazard ratio; TTNT-D, time to first recorded prespecified subsequent systemic therapy or death.

Because each subgroup was independently reconstructed and rematched, subgroup denominators were distinct from the primary cohort (**Figure 4**; **Supplementary Table S3**). Associations were similar by sex (male: HR, 0.802; 95% CI, 0.751–0.857; female: HR, 0.796; 95% CI, 0.740–0.856; P for heterogeneity = 0.881) and age (<65 years: HR, 0.791; 95% CI, 0.706–0.885; 65–74 years: HR, 0.763; 95% CI, 0.704–0.828; ≥75 years: HR, 0.804; 95% CI, 0.745–0.867; P for heterogeneity = 0.647). No statistically significant heterogeneity was detected by metastatic disease (yes: HR, 0.736; 95% CI, 0.681–0.796; no: HR, 0.805; 95% CI, 0.755–0.859; P = 0.083), baseline systemic steroids (yes: HR, 0.793; 95% CI, 0.744–0.846; no: HR, 0.748; 95% CI, 0.692–0.808; P = 0.255), or concomitant chemotherapy (yes: HR, 0.790; 95% CI, 0.739–0.845; no: HR, 0.769; 95% CI, 0.715–0.827; P = 0.593). ICI-class heterogeneity was also not statistically significant (P = 0.204): HRs were 0.760 (95% CI, 0.719–0.803) for PD-1-only, 0.809 (95% CI, 0.713–0.918) for PD-L1-only, and 0.903 (95% CI, 0.740–1.103) for CTLA-4-containing cohorts; the latter estimate was imprecise and centered near the null.

**Figure 4:**
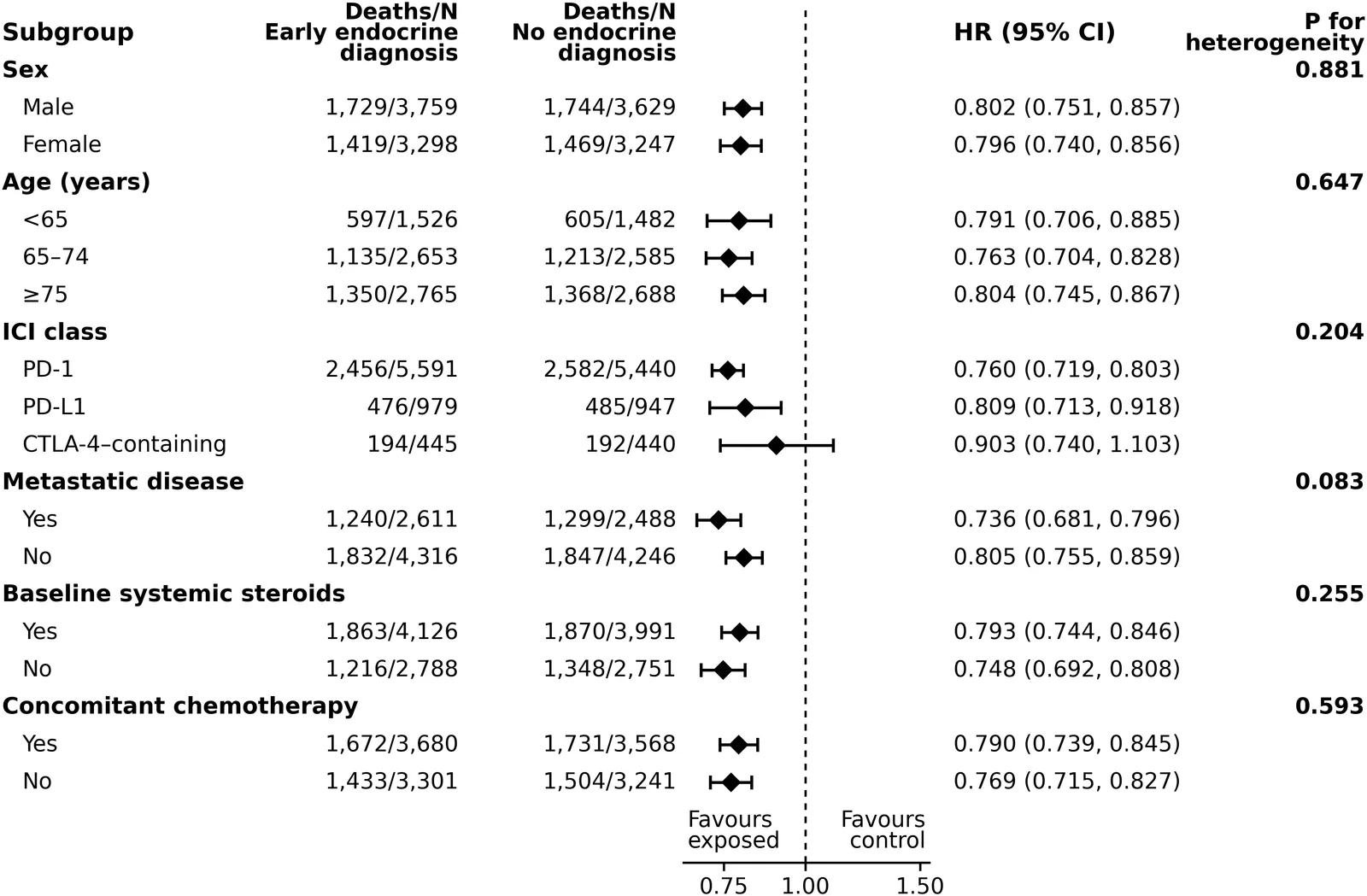
Prespecified subgroup analysis forest plot. Prespecified subgroup analyses of post-landmark OS. Each subgroup cohort was independently reconstructed and independently propensity-score matched; subgroup denominators therefore represent distinct analytic populations and are not subdivisions of the primary matched cohort. ICI classes were mutually exclusive, with CTLA-4-containing exposure assigned priority. HRs and 95% CIs are presented for each stratum. Global P values for heterogeneity were calculated from subgroup-specific log HRs and standard errors derived from their 95% CIs and are summary-level between-stratum tests rather than patient-level Cox interaction terms. CI, confidence interval; CTLA-4, cytotoxic T-lymphocyte–associated protein 4; HR, hazard ratio; ICI, immune checkpoint inhibitor; OS, overall survival; PD-1, programmed cell death 1; PD-L1, programmed death-ligand 1.

All sensitivity analyses remained directionally consistent (**Figure 5**). The HR was 0.757 (95% CI, 0.722–0.793) at the 90-day landmark and 0.761 (95% CI, 0.726–0.798) at the 120-day landmark. Estimates were 0.764 (95% CI, 0.727–0.803) for diagnoses during days 1–90, 0.760 (95% CI, 0.721–0.801) for diagnoses during days 91–180, and 0.767 (95% CI, 0.729–0.808) for the repeat-code definition requiring at least two qualifying group instances (all log-rank P < 0.0001). The interface did not permit confirmation that repeat-code instances occurred on separate dates.

**Figure 5:**
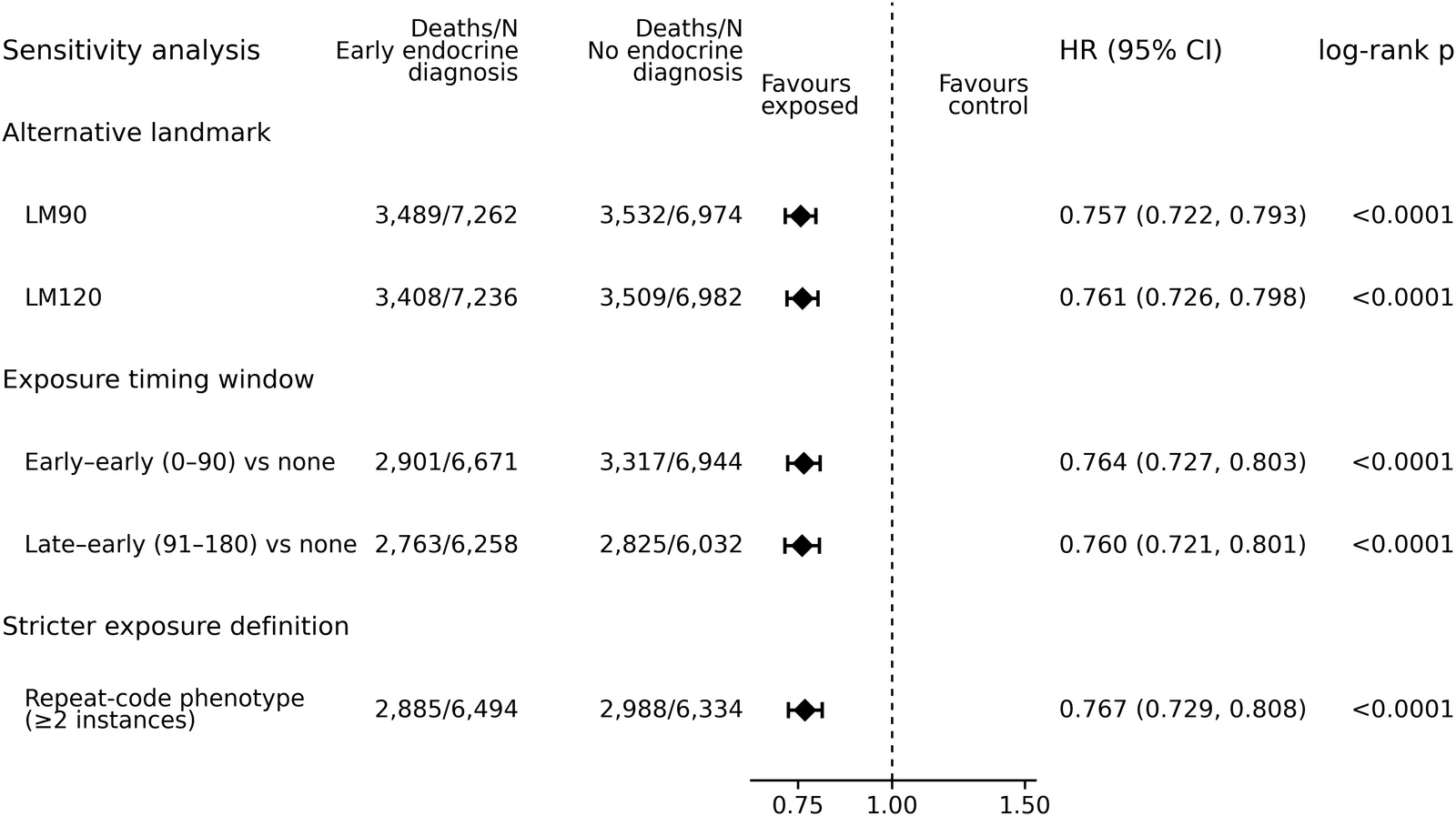
Sensitivity analysis forest plot. Sensitivity analyses of post-landmark OS varied the landmark time (LM90 and LM120), evaluated diagnosis timing during days 1–90 or 91–180 within the 180-day landmark framework, and applied a stricter repeat-code definition requiring at least two qualifying diagnosis-group instances during days 1–180. The LIVE interface did not permit confirmation that repeat-code instances occurred on separate dates. HRs and 95% CIs, death counts, analytic denominators, and log-rank P values are displayed. CI, confidence interval; HR, hazard ratio; LM, landmark; OS, overall survival.

## Discussion

In this federated electronic health record landmark cohort, newly recorded endocrine diagnoses during days 1–180 after first ICI were associated with a 22% lower hazard of subsequent mortality beginning on day 181. The association remained directionally similar across independently reconstructed subgroup analyses and across alternative landmarks, exposure-timing windows, and the stricter repeat-code definition. Because exposure was based on structured diagnosis records rather than clinical adjudication, the finding should be interpreted as a conditional association among patients who satisfied the landmark and observability criteria, not evidence that endocrine toxicity causes improved survival or predicts comparative benefit from ICI. Prior studies of clinically attributed irAEs have reported associations with response and survival, although the magnitude varies by organ system, severity, and analytic design (4, 10–19). Endocrine irAEs are often manageable with hormone replacement and may permit continued immunotherapy (6–9). In NSCLC, thyroid dysfunction during PD-1/PD-L1 blockade and pituitary dysfunction have been associated with favorable outcomes in several cohorts (20–24). These clinical observations provide context for the observed association, but they do not validate diagnosis codes as confirmed irAEs. Our broader code-based definition may include delayed documentation of pre-existing disease, endocrine effects of metastases or radiation, systemic illness, glucocorticoid exposure or withdrawal, and disorders unrelated to ICI. Landmarking separated the 180-day exposure-assessment period from outcome follow-up and therefore addressed the most direct overlap between exposure classification and survival time. Nonetheless, it conditions the analysis on remaining observable near the landmark and cannot eliminate treatment-duration, exposure-opportunity, or surveillance bias. Patients with longer ICI exposure, more frequent laboratory monitoring, or greater healthcare contact may have more opportunities to receive an endocrine diagnosis. Anchoring eligibility at the first recorded ICI administration reduced within-network prevalent-use ambiguity, although treatment before the available record could not be excluded (25). Immortal-time and other time-related biases can materially affect analyses of post-treatment events (5, 26), and landmark methods provide a transparent conditional estimand rather than unconditional causal inference (27, 28). The required visit during months 5–6 was only an operational observability proxy and did not establish uninterrupted enrollment or complete follow-up. Conditioning eligibility on a recorded visit near the landmark may preferentially select patients with ongoing healthcare engagement and introduce additional selection or conditioning bias; the findings therefore apply only to patients meeting this observability requirement. Exposure classification remains a major limitation. The primary definition required one qualifying thyroid, pituitary, or adrenal diagnosis-group instance; it did not require laboratory confirmation, endocrine-directed treatment, severity grading, or clinician attribution. The repeat-code analysis requiring at least two group instances showed a similar estimate, but the interface could not verify that instances occurred on separate dates. Organ-specific counts, cross-organ overlap, and onset distributions were unavailable in the aggregate report. Accordingly, we cannot determine which endocrine component primarily drove the association or quantify misclassification. The revised propensity model used only pre-index variables and improved balance across demographics, metastatic sites, comorbidities, nicotine dependence, steroid use, prior radiation, antineoplastic therapy, and immunosuppression. However, Eastern Cooperative Oncology Group performance status, PD-L1 expression, driver alterations, site-specific histology, quantitative disease burden, exact treatment line, calendar period, and healthcare organization or region could not be reliably incorporated. Residual confounding by prognosis, treatment selection, and intensity of follow-up therefore remains possible. The primary cohort intentionally included heterogeneous real-world ICI settings; independently matched subgroup analyses addressed selected treatment contexts but did not make these settings clinically interchangeable. Although no statistically significant between-class heterogeneity was detected, the CTLA-4-containing estimate was imprecise and centered near the null. The independently matched TTNT-D analysis was directionally concordant with OS. However, the endpoint represented the first recorded prespecified subsequent systemic therapy or death, not radiographic progression or formal reconstruction of treatment line. Real-world treatment-based endpoints capture different clinical constructs and may correlate imperfectly with OS (29–31). Severe infection and healthcare utilization showed higher hazards in the exposed cohort. These exploratory findings may reflect increased monitoring, toxicity management, greater survival opportunity to experience nonfatal events, or residual differences in illness severity. Because formal competing-risk regression was unavailable, these Cox HRs should not be interpreted as cumulative-incidence or Fine–Gray estimates. Death may preclude observation of these nonfatal outcomes, and lower mortality in the endocrine-diagnosis group may increase the opportunity for events or healthcare encounters to be recorded; competing mortality may therefore contribute to the observed associations. Federated electronic health record analyses enable large multicenter cohorts with standardized query logic but are limited by diagnostic-code accuracy, incomplete capture of deaths and care outside participating systems, and absence of patient-level adjudication or export (32). The platform outcome-level exclusion of deaths recorded before day 181 produced outcome-specific OS denominators. The aggregate output did not document whether the Cox models accounted for matched-pair dependence through stratification or cluster-robust variance estimation, and matched-pair identifiers were unavailable for independent evaluation. Accordingly, the reported confidence intervals may not fully reflect dependence introduced by 1:1 matching and should be interpreted cautiously. Sequential, mutually exclusive attrition counts also could not be derived because the platform evaluated complete Boolean and temporal criteria jointly. In conclusion, newly recorded endocrine diagnoses during the first 180 days after ICI initiation were associated with lower subsequent mortality among patients with NSCLC meeting the landmark and observability criteria. Prospective studies incorporating standardized endocrine laboratory testing, medication data, clinical attribution, toxicity grade, treatment duration, and detailed tumor factors are needed to determine the clinical meaning of this association.

## Data Availability

The data analyzed in this study is subject to the following licenses/restrictions: The datasets analyzed in this study are subject to TriNetX access and data-use restrictions. The underlying de-identified patient-level electronic health record data are maintained within the TriNetX Global Collaborative Network and cannot be accessed, downloaded, or distributed by the authors. Only aggregate, de-identified query outputs were analyzed. Access to the underlying data is limited to authorized TriNetX organizations and is governed by applicable licensing, privacy, and data-use agreements. Requests to access these datasets should be directed to TriNetX, LLC https://trinetx.com/contact-us/.

## Supplementary material

Supplementary Figure 1 Covariate balance before and after propensity-score matching. Love plot showing the absolute SMD for each prespecified primary propensity-score covariate before and after matching. The dashed vertical line indicates the prespecified balance threshold of 0.10; all postmatching absolute SMDs were below this threshold. Abbreviations: SMD, standardized mean difference.

Supplementary Figure 2 Propensity-score distributions before and after matching. Platform-generated propensity-score density distributions for the early endocrine diagnosis and no early endocrine diagnosis cohorts before and after 1:1 matching. The postmatching distributions demonstrate close overlap between cohorts.

## References

[1] Reck M Rodríguez-Abreu D Robinson AG Hui R Csőszi T Fülöp A . Pembrolizumab versus chemotherapy for PD-L1-positive non-small-cell lung cancer. N Engl J Med. (2016) 375:1823–33. doi: 10.1056/NEJMoa1606774 27718847

[2] Gandhi L Rodríguez-Abreu D Gadgeel S Esteban E Felip E De Angelis F . Pembrolizumab plus chemotherapy in metastatic non-small-cell lung cancer. N Engl J Med. (2018) 378:2078–92. doi: 10.1056/NEJMoa1801005 29658856

[3] Antonia SJ Villegas A Daniel D Vicente D Murakami S Hui R . Durvalumab after chemoradiotherapy in stage III non-small-cell lung cancer. N Engl J Med. (2017) 377:1919–29. doi: 10.1056/NEJMoa1709937 28885881

[4] Hussaini S Chehade R Boldt RG Raphael J Blanchette P Maleki Vareki S . Association between immune-related side effects and efficacy and benefit of immune checkpoint inhibitors: A systematic review and meta-analysis. Cancer Treat Rev. (2021) 92:102134. doi: 10.1016/j.ctrv.2020.102134 33302134

[5] Suissa S . Immortal time bias in pharmaco-epidemiology. Am J Epidemiol. (2008) 167:492–9. doi: 10.1093/aje/kwm324 18056625

[6] Brahmer JR Lacchetti C Schneider BJ Atkins MB Brassil KJ Caterino JM . Management of immune-related adverse events in patients treated with immune checkpoint inhibitor therapy: American Society of Clinical Oncology clinical practice guideline. J Clin Oncol. (2018) 36:1714–68. doi: 10.1200/JCO.2017.77.6385 29442540 PMC6481621

[7] Haanen JBAG Carbonnel F Robert C Kerr KM Peters S Larkin J . Management of toxicities from immunotherapy: ESMO Clinical Practice Guidelines for diagnosis, treatment and follow-up. Ann Oncol. (2017) 28:iv119–42. doi: 10.1093/annonc/mdx225 28881921

[8] Puzanov I Diab A Abdallah K Bingham CO Brogdon C Dadu R . Managing toxicities associated with immune checkpoint inhibitors: Consensus recommendations from the Society for Immunotherapy of Cancer (SITC) Toxicity Management Working Group. J Immunother Cancer. (2017) 5:95. doi: 10.1186/s40425-017-0300-z 29162153 PMC5697162

[9] Barroso-Sousa R Barry WT Garrido-Castro AC Hodi FS Min L Krop IE . Incidence of endocrine dysfunction following the use of different immune checkpoint inhibitor regimens: A systematic review and meta-analysis. JAMA Oncol. (2018) 4:173–82. doi: 10.1001/jamaoncol.2017.3064 28973656 PMC5838579

[10] Das S Johnson DB . Immune-related adverse events and anti-tumor efficacy of immune checkpoint inhibitors. J Immunother Cancer. (2019) 7:306. doi: 10.1186/s40425-019-0805-8 31730012 PMC6858629

[11] Haratani K Hayashi H Chiba Y Kudo K Yonesaka K Kato R . Association of immune-related adverse events with nivolumab efficacy in non-small-cell lung cancer. JAMA Oncol. (2018) 4:374–8. doi: 10.1001/jamaoncol.2017.2925 28975219 PMC6583041

[12] Sato K Akamatsu H Murakami E Sasaki S Kanai K Hayata A . Correlation between immune-related adverse events and efficacy in non-small cell lung cancer treated with nivolumab. Lung Cancer. (2018) 115:71–4. doi: 10.1016/j.lungcan.2017.11.019 29290265

[13] Teraoka S Fujimoto D Morimoto T Kawachi H Ito M Sato Y . Early immune-related adverse events and association with outcome in advanced non-small cell lung cancer patients treated with nivolumab: A prospective cohort study. J Thorac Oncol. (2017) 12:1798–805. doi: 10.1016/j.jtho.2017.08.022 28939128

[14] Ricciuti B Genova C De Giglio A Bassanelli M Dal Bello MG Metro G . Impact of immune-related adverse events on survival in patients with advanced non-small cell lung cancer treated with nivolumab: Long-term outcomes from a multi-institutional analysis. J Cancer Res Clin Oncol. (2019) 145:479–85. doi: 10.1007/s00432-018-2805-3 30506406 PMC11810236

[15] Grangeon M Tomasini P Chaleat S Jeanson A Souquet-Bressand M Khobta N . Association between immune-related adverse events and efficacy of immune checkpoint inhibitors in non-small-cell lung cancer. Clin Lung Cancer. (2019) 20:201–7. doi: 10.1016/j.cllc.2018.10.002 30442524

[16] Chen X Nie J Dai L Hu W Zhang J Han J . Immune-related adverse events and their association with the effectiveness of PD-1/PD-L1 inhibitors in non-small cell lung cancer: A real-world study from China. Front Oncol. (2021) 11:607531. doi: 10.3389/fonc.2021.607531 33747922 PMC7973369

[17] Cook S Samuel V Meyers DE Stukalin I Litt I Sangha R . Immune-related adverse events and survival among patients with metastatic NSCLC treated with immune checkpoint inhibitors. JAMA Netw Open. (2024) 7:e2352302. doi: 10.1001/jamanetworkopen.2023.52302 38236598 PMC10797458

[18] Socinski MA Jotte RM Cappuzzo F Nishio M Mok TSK Reck M . Association of immune-related adverse events with efficacy of atezolizumab in patients with non-small cell lung cancer: Pooled analyses of the phase 3 IMpower130, IMpower132, and IMpower150 randomized clinical trials. JAMA Oncol. (2023) 9:527–35. doi: 10.1001/jamaoncol.2022.7711 36795388 PMC9936386

[19] Lisberg A Tucker DA Goldman JW Wolf B Carroll J Hardy A . Treatment-related adverse events predict improved clinical outcome in NSCLC patients on KEYNOTE-001 at a single center. Cancer Immunol Res. (2018) 6:288–94. doi: 10.1158/2326-6066.CIR-17-0063 29382669 PMC6066474

[20] Osorio JC Ni A Chaft JE Pollina R Kasler MK Stephens D . Antibody-mediated thyroid dysfunction during T-cell checkpoint blockade in patients with non-small-cell lung cancer. Ann Oncol. (2017) 28:583–9. doi: 10.1093/annonc/mdw640 27998967 PMC5834017

[21] Kim HI Kim M Lee SH Park SY Kim YN Kim H . Development of thyroid dysfunction is associated with clinical response to PD-1 blockade treatment in patients with advanced non-small cell lung cancer. Oncoimmunology. (2017) 7:e1375642. doi: 10.1080/2162402X.2017.1375642 29296533 PMC5739550

[22] Thuillier P Joly C Alavi Z Crouzeix G Descourt R Quere G . Thyroid dysfunction induced by immune checkpoint inhibitors is associated with a better progression-free survival and overall survival in non-small cell lung cancer: An original cohort study. Cancer Immunol Immunother. (2021) 70:2023–33. doi: 10.1007/s00262-020-02802-6 33423089 PMC10991310

[23] Zhou Y Xia R Xiao H Pu D Long Y Ding Z . Thyroid function abnormality induced by PD-1 inhibitors have a positive impact on survival in patients with non-small cell lung cancer. Int Immunopharmacol. (2021) 91:107296. doi: 10.1016/j.intimp.2020.107296 33360368

[24] Kobayashi T Iwama S Yasuda Y Okada N Okuji T Ito M . Pituitary dysfunction induced by immune checkpoint inhibitors is associated with better overall survival in both Malignant melanoma and non-small cell lung carcinoma: A prospective study. J Immunother Cancer. (2020) 8:e000779. doi: 10.1136/jitc-2020-000779 32606047 PMC7328763

[25] Ray WA . Evaluating medication effects outside of clinical trials: New-user designs. Am J Epidemiol. (2003) 158:915–20. doi: 10.1093/aje/kwg231 14585769

[26] Lévesque LE Hanley JA Kezouh A Suissa S . Problem of immortal time bias in cohort studies: Example using statins for preventing progression of diabetes. BMJ. (2010) 340:b5087. doi: 10.1136/bmj.b5087 20228141

[27] Anderson JR Cain KC Gelber RD . Analysis of survival by tumor response. J Clin Oncol. (1983) 1:710–9. doi: 10.1200/JCO.1983.1.11.710 6668489

[28] Dafni U . Landmark analysis at the 25-year landmark point. Circ Cardiovasc Qual Outcomes. (2011) 4:363–71. doi: 10.1161/CIRCOUTCOMES.110.957951 21586725

[29] Stewart M Norden AD Dreyer N Henk HJ Abernethy AP Chrischilles E . An exploratory analysis of real-world end points for assessing outcomes among immunotherapy-treated patients with advanced non-small-cell lung cancer. JCO Clin Cancer Inform. (2019) 3:1–15. doi: 10.1200/CCI.18.00155 31335166 PMC6873914

[30] Walker B Boyd M Aguilar K Davies K Espirito J Frytak J . Comparisons of real-world time-to-event end points in oncology research. JCO Clin Cancer Inform. (2021) 5:45–6. doi: 10.1200/CCI.20.00125 33411622

[31] Kehl KL Riely GJ Lepisto EM Lavery JA Warner JL LeNoue-Newton ML . Correlation between surrogate end points and overall survival in a multi-institutional clinicogenomic cohort of patients with non-small cell lung or colorectal cancer. JAMA Netw Open. (2021) 4:e2117547. doi: 10.1001/jamanetworkopen.2021.17547 34309669 PMC8314138

[32] Stein E Hüser M Amirian ES Palchuk MB Brown JS . TriNetX Dataworks-USA: Overview of a multi-purpose, de-identified, federated electronic health record real-world data and analytics network and comparison to the US census. Pharmacoepidemiol Drug Saf. (2025) 34:e70198. doi: 10.1002/pds.70198 40915660 PMC12414656

