# Supplementary material for: Early endocrine diagnoses and subsequent survival after immune checkpoint inhibitor initiation in non-small cell lung cancer: a 180-day landmark cohort study
Source: Front Oncol. 2026 Sep 17;16:1957383. doi: 10.3389/fonc.2026.1957383 (PMC13629181; doi:10.3389/fonc.2026.1957383)
Supplement: Supplementary file 1 [file Table1.docx]

STROBE Cohort Checklist — Completed Cross-reference

Completed for the current Frontiers in Oncology manuscript | Official source: https://www.strobe-statement.org/checklists/

Completion status: all substantive fields populated; no unexplained blanks

# Completion approach

Each item is mapped to the currently saved manuscript or retained study documentation. The author-confirmed rationale for asymmetric baseline lookbacks and the evidence-bounded interpretation of TriNetX stage attrition are stated explicitly; unsupported causation is labelled as inference.

# Summary

All 34 items have an explicit evidence-based response. Author-confirmed design rationale and source reconciliation are recorded, and residual uncertainty is labelled explicitly. No response cell is blank.

| **Item** | **Topic** | **Recommendation** | **Reported on page / location** | **Assessment / evidence** |
| --- | --- | --- | --- | --- |
| 1(a) | Title/abstract | Indicate the study design with a commonly used term in the title or abstract. | p. 1, title and Abstract | COMPLETE — the title identifies a time-aligned landmark cohort, and the Abstract identifies a target-trial-informed retrospective cohort. |
| 1(b) | Title/abstract | Provide an informative, balanced summary of methods and findings. | p. 1, Abstract | COMPLETE — design, participants, exposure, matching, outcomes, principal estimates, and the conclusion are summarized. |
| 2 | Background | Explain the scientific background and rationale. | pp. 1–2, Introduction | COMPLETE — the clinical rationale, inconsistent prior evidence, and risk of guarantee-time bias are explained. |
| 3 | Objectives | State specific objectives, including prespecified hypotheses. | p. 2, end of Introduction | PARTIALLY REPORTED — the objective is explicit, but the manuscript does not identify a prespecified hypothesis. |
| 4 | Study design | Present key elements of study design early. | p. 1, Abstract; pp. 2–3, Methods | COMPLETE — retrospective cohort design, first-ICI index, exposure-classification window, day-180 landmark, and 1:1 PSM are stated early. |
| 5 | Setting | Describe setting, locations and relevant calendar dates, including exposure and follow-up. | pp. 2–3, Methods; retained query report (18 Feb 2026) | PARTIALLY REPORTED — the global network and relative study windows are stated; the manuscript omits the 1 Jan 2010–31 Jan 2026 cohort-entry period, 18 Feb 2026 query date, and geographic distribution. |
| 6(a) | Participants | Give eligibility, selection and follow-up methods. | pp. 2–3, Methods; p. 10, Figure 1 legend | PARTIALLY REPORTED — NSCLC/ICI eligibility, the first-ICI index, EirAE classification, landmarking, and unequal baseline lookbacks are described. AUTHOR-CONFIRMED RATIONALE: the exposed cohort used a 1-year pre-index exclusion because only a shorter baseline period was considered necessary to exclude clinically apparent pre-existing EirAEs before identifying a new early event; the control cohort intentionally used a stricter 5-year exclusion to minimize latent or pre-existing endocrine diagnoses and reduce control-group misclassification. Small-cell exclusion, the required post-ICI visit, and other selection details are not fully reported. |
| 6(b) | Participants | For matched studies, give matching criteria and exposed/unexposed numbers. | p. 3, Methods/Results; p. 9, Table 1 | COMPLETE — 1:1 logistic-propensity-score greedy nearest-neighbor matching without replacement, a 0.1-SD caliper, matched covariates, and group sizes are given. |
| 7 | Variables | Define outcomes, exposures, predictors, confounders and effect modifiers. | pp. 2–3, Methods; retained query documentation | PARTIALLY REPORTED — main exposures, outcomes, covariates, and subgroup factors are defined and Boolean-OR composite logic is explained; complete code sets and validation are not included in the submitted manuscript. |
| 8 | Data sources | Give data sources and assessment methods for each variable. | pp. 2–3, Data source and Outcomes | PARTIALLY REPORTED — TriNetX routine-EHR domains and aggregate analysis are described, but variable-specific ascertainment algorithms and death-capture details are incomplete. |
| 9 | Bias | Describe efforts to address potential sources of bias. | pp. 1–3; p. 5, Discussion | PARTIALLY REPORTED — day-180 landmark alignment addresses guarantee-time bias and PSM addresses measured confounding. The author confirms that the 1-year/5-year lookback asymmetry was intentional to improve control specificity; nevertheless, it may cause differential ascertainment or selection bias. Coding, external-death capture, missingness, and residual confounding remain relevant. |
| 10 | Study size | Explain how the study size was arrived at. | p. 3, Results; p. 9, Table 1; IRB protocol; retained query report | NOT REPORTED — the manuscript gives achieved cohort sizes but no sample-size rationale or a priori calculation. The protocol anticipated approximately 7,000 per cohort without a formal power calculation; available data yielded 6,457 matched participants per group. |
| 11 | Quantitative variables | Explain handling and grouping of quantitative variables and why. | p. 3, Methods; p. 9, Table 1; Figure 4 | PARTIALLY REPORTED — age is continuous for baseline description/matching and grouped as <65, 65–74, and ≥75 years for subgroup analysis; the grouping rationale is not stated. |
| 12(a) | Statistics | Describe all statistical methods, including confounding control. | p. 3, Methods; p. 9, Table 1; Figures 2–5 | PARTIALLY REPORTED — propensity-score matching, SMD balance, Kaplan–Meier, log-rank, Cox models, HRs, and 95% CIs are described; software/analysis date, censoring, and proportional-hazards assessment are omitted. |
| 12(b) | Statistics | Describe subgroup and interaction methods. | p. 3, Methods; pp. 3–4, Results; Figure 4 | PARTIALLY REPORTED — prespecified stratified analyses are described and reported; no formal interaction test or multiplicity approach is documented. |
| 12(c) | Statistics | Explain how missing data were addressed. | Not reported in the current manuscript; retained aggregate export | NOT REPORTED — no missing-data description, imputation method, or complete-case rule is documented. Variable-level missingness was unavailable from the aggregate export; this does not establish that missing data were absent. |
| 12(d) | Statistics | Explain how loss to follow-up was addressed. | p. 3, outcome start only; retained query report | NOT REPORTED — the manuscript states that outcome assessment begins at day 180 but does not describe loss-to-follow-up handling. The retained TriNetX report specifies days 180–1,825 and censoring after the last recorded fact. |
| 12(e) | Statistics | Describe sensitivity analyses. | p. 3, Methods; p. 4, Results; Figure 5 | COMPLETE — 90- and 120-day landmarks and alternative exposure-timing definitions assess robustness. |
| 13(a) | Participants | Report numbers at each study stage. | p. 3, Results; p. 9, Table 1; retained query report | PARTIALLY REPORTED — query-defined cohorts were 7,905/23,574; TriNetX-labelled pre-matching analysis populations were 6,458/19,655; matched groups were 6,457/6,457. The export does not state why query-defined counts fell before PSM. The most plausible interpretation is formation of an analysis-eligible propensity-score covariate matrix, potentially excluding records with unavailable index/covariate values (especially Age at Index), but this is an inference rather than a platform-confirmed cause. |
| 13(b) | Participants | Give reasons for non-participation at each stage. | pp. 2–3; p. 10, Figure 1 legend | PARTIALLY REPORTED — principal query exclusions are named. The initial reductions were 1,447 (18.3%) in the exposed cohort and 3,919 (16.6%) in controls before the TriNetX-labelled pre-matching populations. Outcome-specific prior-window exclusions, HCO response counts, the required post-ICI visit, the >20-year index exclusion, and PSM itself do not account for this stage. No platform-confirmed reason is available. |
| 13(c) | Participants | Consider a flow diagram. | Figure 1 and p. 10 legend | COMPLETE — a cohort-assembly, landmark, and matching flow diagram is provided; incomplete counts/reasons are recorded under Items 13(a) and 13(b). |
| 14(a) | Descriptive data | Give participant characteristics, exposures and confounders. | p. 9, Table 1 | COMPLETE — pre- and post-match demographics, race, comorbidities, metastatic sites, and SMDs are tabulated. |
| 14(b) | Descriptive data | Indicate missing data for each variable. | Not reported in the current manuscript or Table 1 | NOT REPORTED — variable-specific missing/unknown counts are not given and some displayed categories do not sum to cohort totals. The aggregate export did not provide a missing-data algorithm; no investigator-level imputation was performed. |
| 14(c) | Descriptive data | Summarize follow-up time. | Not reported in the current manuscript; retained query report | NOT REPORTED — median and total follow-up by cohort are absent. The retained report gives only the configured day-180–1,825 window and censoring after the last recorded fact. |
| 15 | Outcome data | Report outcome events or summary measures over time. | pp. 3–4, Results; Figures 2–5 and p. 10 legends | PARTIALLY REPORTED — matched HRs/Kaplan–Meier curves and secondary-outcome event counts are reported; OS event counts are absent. SOURCE RECONCILED: the retained TriNetX output confirms 698/1,519 exposed TTNT-D events (control 605/1,357; HR 0.853, 95% CI 0.765–0.951; P=0.0041). Figure 3 has been corrected; the author will update the manuscript text, which still contains 688/1,519. |
| 16(a) | Main results | Give crude/adjusted estimates, precision and confounders. | pp. 3–4, Results; p. 9, Table 1; Figures 2–5 | PARTIALLY REPORTED — matched HRs, 95% CIs, balance diagnostics, and adjustment variables are reported; crude/unmatched estimates and the rationale for covariate selection are not provided. |
| 16(b) | Main results | Report category boundaries for continuous variables. | p. 3, Methods; p. 4, Results; Figure 4 | COMPLETE — age-category boundaries are <65, 65–74, and ≥75 years. |
| 16(c) | Main results | When relevant, translate relative risk into absolute risk over a meaningful period. | pp. 3–4, Results; Figures 2–5 | NOT APPLICABLE — the prespecified estimand is a conditional post-landmark time-to-event HR rather than an absolute causal risk; Kaplan–Meier curves display absolute survival probability over time. |
| 17 | Other analyses | Report subgroup, interaction and sensitivity analyses. | pp. 3–4, Results; Figures 4–5 | COMPLETE — prespecified subgroup and timing/landmark sensitivity analyses are presented with HRs and 95% CIs; interaction testing is addressed under Item 12(b). |
| 18 | Key results | Summarize key results with reference to objectives. | p. 1, Abstract; pp. 3–5, Results/Discussion | COMPLETE — the key conditional survival result, modest effect size, secondary morbidity/utilization findings, and robustness analyses are tied to the objective. |
| 19 | Limitations | Discuss limitations and direction/magnitude of bias. | p. 5, Discussion | PARTIALLY REPORTED — diagnostic coding, incomplete out-of-system death capture, limited tumor/treatment variables, and residual confounding are discussed. The intentional 1-year/5-year lookback asymmetry was intended to reduce control misclassification, but it can introduce differential ascertainment or selection; its direction and magnitude cannot be established from the aggregate export. |
| 20 | Interpretation | Give a cautious overall interpretation considering objectives, limitations and other evidence. | pp. 4–5, Discussion | COMPLETE — interpretation is cautious and considers observational design, causal uncertainty, prior evidence, outcome limitations, and the need for prospective confirmation. |
| 21 | Generalisability | Discuss external validity. | p. 2, Data source; p. 5, Discussion | PARTIALLY REPORTED — the large multi-organization network is described, but geographic/system coverage and explicit limits on transportability are not discussed. |
| 22 | Funding | Give funding sources and role of funders. | p. 6, Funding | COMPLETE AS CURRENTLY REPORTED — the institutional project number, funder role, and absence of article-processing-charge support are stated. |
