# Supplementary material for: Early endocrine diagnoses and subsequent survival after immune checkpoint inhibitor initiation in non-small cell lung cancer: a 180-day landmark cohort study
Source: Front Oncol. 2026 Sep 17;16:1957383. doi: 10.3389/fonc.2026.1957383 (PMC13629181; doi:10.3389/fonc.2026.1957383)
Supplement: Supplementary file 2 [file Table2.docx]

RECORD Checklist — Completed Cross-reference

Completed for the current Frontiers in Oncology manuscript | Official source: https://www.record-statement.org/checklist.php

Completion status: all substantive fields populated; no unexplained blanks

# Completion approach

Each item is mapped to the currently saved manuscript or retained study documentation. The author-confirmed rationale for asymmetric baseline lookbacks and the evidence-bounded interpretation of TriNetX stage attrition are stated explicitly; unsupported causation is labelled as inference.

# Summary

All 13 extension items have an explicit evidence-based response. Author-confirmed design rationale and platform-stage limitations are recorded, and residual uncertainty is labelled explicitly. No response cell is blank.

| **Item** | **Topic** | **Recommendation** | **Reported on page / location** | **Assessment / evidence** |
| --- | --- | --- | --- | --- |
| 1.1 | Title/abstract | State the routinely collected data type and database name. | p. 1, Abstract; p. 2, Data source | PARTIALLY REPORTED — EHR data are identified in the Abstract; the TriNetX Global Collaborative Network is named only in Methods, not in the title or Abstract. |
| 1.2 | Title/abstract | State geographic region and timeframe. | p. 2, Data source; retained query report (18 Feb 2026) | PARTIALLY REPORTED — global scope is stated in Methods; the exact 1 Jan 2010–31 Jan 2026 cohort-entry period and 18 Feb 2026 query date appear only in the retained query report. |
| 1.3 | Title/abstract | If databases were linked, state this and linkage level. | p. 2, Data source; retained query documentation | NOT APPLICABLE — one federated TriNetX network was queried; investigators performed no external database or patient-level record linkage. |
| 6.1 | Participants | Describe detailed population-selection codes/algorithms. | pp. 2–3, Methods; retained query documentation | PARTIALLY REPORTED — principal first-ICI, NSCLC, EirAE, landmark, and exclusion logic is described. AUTHOR-CONFIRMED RATIONALE: the exposed cohort used a 1-year pre-index exclusion because only a shorter baseline period was considered necessary to exclude clinically apparent pre-existing EirAEs before identifying a new early event; controls intentionally used a stricter 5-year exclusion to minimize latent or pre-existing endocrine diagnoses and reduce control misclassification. Complete executable code lists and the comparator query's nonuniform Boolean condition are not reproduced in the manuscript. |
| 6.2 | Participants | Describe validation of population-selection codes/algorithms. | p. 5, Discussion; retained query documentation | NOT PERFORMED — no study-specific chart review, adjudication, or independent phenotype/code validation is documented; potential code-based misclassification is acknowledged. |
| 6.3 | Participants | If linkage was used, give a linkage flow diagram. | Not applicable — no database linkage performed | NOT APPLICABLE — a linkage flow diagram is unnecessary because the study used no linked external databases or investigator-level linkage. |
| 7.1 | Variables | List complete codes/algorithms for exposures, outcomes, confounders and modifiers. | pp. 2–3, Methods; retained query documentation | PARTIALLY REPORTED — principal exposure, outcome, exclusion, covariate, and Boolean-composite logic is described; full code lists and executable queries are retained locally but are not fully included in the submitted manuscript or a public repository. |
| 12.1 | Statistics | Describe investigator access to the database population. | p. 2, Data source; p. 6, Data Availability | COMPLETE — investigators queried the LIVE platform and received only deidentified aggregate results; no patient-level review or export occurred. |
| 12.2 | Statistics | Describe data cleaning methods. | p. 2, Data source; retained platform workflow | NO INVESTIGATOR-LEVEL CLEANING PERFORMED — only aggregate TriNetX outputs were accessible; the retained export does not document additional investigator deduplication, plausibility checks, record editing, or missing-data procedures. |
| 12.3 | Statistics | Describe linkage methods and quality evaluation. | Not applicable — no database linkage performed | NOT APPLICABLE — linkage methods and quality metrics do not apply because no record-level linkage was conducted. |
| 13.1 | Participants | Report detailed database filtering and attrition. | p. 3, Results; p. 9, Table 1; Figure 1; retained query report | PARTIALLY REPORTED — query-defined cohorts were 7,905/23,574, TriNetX-labelled pre-matching analysis populations were 6,458/19,655, and matched cohorts were 6,457/6,457. The initial reductions were 1,447 (18.3%) and 3,919 (16.6%). The export does not state the cause. Formation of an analysis-eligible propensity-score covariate matrix, potentially excluding records with unavailable index/covariate values (especially Age at Index), is the most plausible explanation but remains an inference, not a platform-confirmed fact. |
| 19.1 | Limitations | Discuss implications of non-purpose-collected data. | p. 5, Discussion | PARTIALLY REPORTED — routine-care coding, incomplete external-death capture, limited clinical granularity, and residual confounding are discussed. The author confirms that the exposed 1-year versus control 5-year baseline lookback was intentional to improve control specificity; the asymmetry can nevertheless introduce differential ascertainment or selection bias. Unavailable missingness measures and temporal/network changes are not explicit. |
| 22.1 | Accessibility | State access to protocol, raw data and programming code. | p. 6, Data Availability; retained query documentation | PARTIALLY REPORTED — the Data Availability statement explains restricted TriNetX access and aggregate-only author access; no public protocol, statistical analysis plan, cohort-query repository, or reusable analysis-code URL is documented. |
