# Supplementary material for: Early endocrine diagnoses and subsequent survival after immune checkpoint inhibitor initiation in non-small cell lung cancer: a 180-day landmark cohort study
Source: Front Oncol. 2026 Sep 17;16:1957383. doi: 10.3389/fonc.2026.1957383 (PMC13629181; doi:10.3389/fonc.2026.1957383)
Supplement: Supplementary file 3 [file Table3.docx]

**Supplementary Table S1. Operational definition of the early endocrine diagnosis phenotype.**

**Panel A. Qualifying diagnosis concepts.**

| **Organ system** | **ICD-10-CM code** | **Concept description** |
| --- | --- | --- |
| Thyroid | E03 | Other hypothyroidism |
| Thyroid | E05 | Thyrotoxicosis [hyperthyroidism] |
| Thyroid | E06 | Thyroiditis |
| Pituitary | E23 | Hypofunction and other disorders of the pituitary gland |
| Adrenal | E27 | Other disorders of adrenal gland |
| Adrenal | E27.1 | Primary adrenocortical insufficiency |
| Adrenal | E27.3 | Drug-induced adrenocortical insufficiency |
| Adrenal | E27.4 | Other and unspecified adrenocortical insufficiency |

All concepts were selected from the diagnosis domain using ICD-10-CM coding. Codes and included descendants were applied as specified in the locked platform query.

**Panel B. Query implementation.**

| **Element** | **Operational definition** |
| --- | --- |
| Index anchor | First recorded immune checkpoint inhibitor (ICI) administration. |
| Common baseline exclusion | Both cohorts excluded any qualifying endocrine diagnosis recorded during days -365 to -1 relative to the ICI index. |
| Primary exposed cohort | At least 1 qualifying endocrine diagnosis-group instance during days 1-180 after ICI initiation. |
| Primary control cohort | No qualifying endocrine diagnosis-group instance during days 1-180 after ICI initiation. |
| Stricter phenotype sensitivity | At least 2 qualifying diagnosis-group instances during days 1-180 after ICI initiation. The LIVE interface did not permit confirmation that the instances occurred on separate dates. |
| Medication/laboratory confirmation | Not required in the primary phenotype. No clinician adjudication, endocrine-directed medication requirement, or laboratory-confirmation requirement was applied. |
| Terminology in revised manuscript | The code-based exposure is described as an early endocrine diagnosis phenotype rather than a confirmed endocrine immune-related adverse event. |

The locked primary platform report provides the code definitions and temporal rules but does not provide organ-specific patient counts, cross-organ overlap, or diagnosis-onset distributions. These quantities were not inferred from the aggregate report.
