# Supplementary material for: Early endocrine diagnoses and subsequent survival after immune checkpoint inhibitor initiation in non-small cell lung cancer: a 180-day landmark cohort study
Source: Front Oncol. 2026 Sep 17;16:1957383. doi: 10.3389/fonc.2026.1957383 (PMC13629181; doi:10.3389/fonc.2026.1957383)
Supplement: Supplementary file 4 [file Table4.docx]

**Supplementary Table S2. Prespecified propensity-score covariates and covariate balance in the primary 180-day landmark cohort.**

The primary analysis included 7,696 exposed and 22,273 control patients before matching and 7,696 patients in each cohort after 1:1 propensity-score matching. All coding-based covariates were ascertained during days -365 to -1; age was measured at index and sex/race were obtained from the demographic record.

| **Domain** | **Covariate (coding system; code)** | **Ascertainment window** | **Absolute SMD before matching** | **Absolute SMD after matching** |
| --- | --- | --- | --- | --- |
| Demographic | Age at index (TriNetX demographic; AI) | Index date | 0.015 | 0.004 |
| Demographic | White (CDC race code; 2106-3) | Demographic record | 0.269 | 0.002 |
| Demographic | Female (TriNetX demographic; F) | Demographic record | 0.103 | 0.005 |
| Demographic | Black or African American (CDC race code; 2054-5) | Demographic record | 0.012 | <0.001 |
| Demographic | Male (TriNetX demographic; M) | Demographic record | 0.103 | 0.005 |
| Demographic | Asian (CDC race code; 2028-9) | Demographic record | 0.098 | 0.013 |
| Diagnosis | Secondary malignant neoplasm of liver and intrahepatic bile duct (ICD-10-CM; C78.7) | Days -365 to -1 | 0.011 | 0.010 |
| Diagnosis | Secondary malignant neoplasm of bone (ICD-10-CM; C79.51) | Days -365 to -1 | 0.042 | 0.004 |
| Diagnosis | Secondary malignant neoplasm of brain (ICD-10-CM; C79.31) | Days -365 to -1 | 0.084 | 0.018 |
| Diagnosis | Diabetes mellitus (ICD-10-CM; E08-E13) | Days -365 to -1 | 0.011 | 0.021 |
| Diagnosis | Other chronic obstructive pulmonary disease (ICD-10-CM; J44) | Days -365 to -1 | 0.052 | 0.006 |
| Diagnosis | Chronic kidney disease (ICD-10-CM; N18) | Days -365 to -1 | 0.010 | 0.041 |
| Diagnosis | Heart failure (ICD-10-CM; I50) | Days -365 to -1 | 0.001 | 0.025 |
| Diagnosis | Nicotine dependence (ICD-10-CM; F17) | Days -365 to -1 | 0.101 | 0.011 |
| Diagnosis | Long-term (current) use of systemic steroids (ICD-10-CM; Z79.52) | Days -365 to -1 | 0.034 | 0.035 |
| Diagnosis/procedure proxy | Encounter for antineoplastic radiation therapy (ICD-10-CM; Z51.0) | Days -365 to -1 | 0.096 | 0.027 |
| Medication | Antineoplastic agents (ATC; L01) | Days -365 to -1 | 0.026 | 0.032 |
| Medication | Immunosuppressants (ATC; L04A) | Days -365 to -1 | 0.008 | 0.027 |

Abbreviations: ATC, Anatomical Therapeutic Chemical classification; SMD, standardized mean difference. The prespecified balance threshold was an absolute SMD <0.10; all postmatching covariates met this threshold. The corresponding Love plot and propensity-score overlap distributions are presented in Supplementary Figures S1 and S2.
