# Supplementary material for: Early endocrine diagnoses and subsequent survival after immune checkpoint inhibitor initiation in non-small cell lung cancer: a 180-day landmark cohort study
Source: Front Oncol. 2026 Sep 17;16:1957383. doi: 10.3389/fonc.2026.1957383 (PMC13629181; doi:10.3389/fonc.2026.1957383)
Supplement: Supplementary file 5 [file Table5.docx]

**Supplementary Table S3. Subgroup-specific cohort construction, matching, balance, and overall-survival estimates.**

**Panel A. Independently reconstructed subgroup queries.**

| **Subgroup variable** | **Level** | **Subgroup restriction applied before matching** | **Analysis name** | **Report generated (UTC)** |
| --- | --- | --- | --- | --- |
| Sex | Male | Male demographic record | SG_M | 11:33:08 |
| Sex | Female | Female demographic record | SG_F | 11:36:39 |
| Age | <65 years | Age at index <65 years | SG_<65y | 13:59:10 |
| Age | 65-74 years | Age at index 65-74 years | SG_65-74y | 14:02:54 |
| Age | >=75 years | Age at index >=75 years | SG_>75y | 14:06:01 |
| ICI class | PD-1 only | PD-1 inhibitor required; PD-L1 and CTLA-4 inhibitors excluded within 1 month on/after first ICI | SG_PD1 | 14:16:20 |
| ICI class | PD-L1 only | PD-L1 inhibitor required; PD-1 and CTLA-4 inhibitors excluded within 1 month on/after first ICI | SG_PDL1 | 14:18:45 |
| ICI class | CTLA-4-containing | CTLA-4 inhibitor required; concomitant PD-1/PD-L1 exposure permitted | SG_CTLA4 containing | 14:22:25 |
| Metastatic disease | Yes | At least 1 prespecified secondary malignant-neoplasm code (C78.7, C79.51, C79.31, C78.2, or C79.3) | SG_Metastasis+ | 14:25:10 |
| Metastatic disease | No | No prespecified secondary malignant-neoplasm code (C78.7, C79.51, C79.31, C78.2, or C79.3) | SG_Metastasis- | 14:31:51 |
| Baseline systemic steroids | Yes | Recorded systemic steroid use within 3 months and 1 day before first ICI | SG_Steroids+ | 14:35:21 |
| Baseline systemic steroids | No | No recorded systemic steroid use within 3 months and 1 day before first ICI | SG_Steroids- | 14:38:41 |
| Concomitant chemotherapy | Yes | Prespecified chemotherapy recorded within 1 month before to 1 month after first ICI | SG_ICI+CT | 14:46:00 |
| Concomitant chemotherapy | No | No prespecified chemotherapy within 1 month before to 1 month after first ICI | SG_ICI-CT | 14:47:48 |

All subgroup reports were generated on August 16, 2026, using the Global Collaborative Network. Each subgroup was independently reconstructed and propensity-score matched because the aggregate LIVE interface does not permit patient-level stratification of an existing matched cohort.

**Panel B. Subgroup-specific analytic populations, balance, and overall-survival estimates.**

| **Subgroup / level** | **Cohort sizes, exposed / control Query; pre-match; matched** | **Deaths/N, exposed / control** | **HR (95% CI)** | **Log-rank P** | **Maximum postmatch absolute SMD** | **P for heterogeneity** |
| --- | --- | --- | --- | --- | --- | --- |
| Sex: Male | Query: 4,641/14,327 Pre-match: 4,055/12,871 Matched: 4,054/4,054 | 1,729/3,759 1,744/3,629 | 0.802 (0.751-0.857) | <0.0001 | 0.045 | 0.881 |
| Sex: Female | Query: 3,890/9,994 Pre-match: 3,562/9,175 Matched: 3,560/3,560 | 1,419/3,298 1,469/3,247 | 0.796 (0.740-0.856) | <0.0001 | 0.047 |  |
| Age: <65 years | Query: 1,802/5,498 Pre-match: 1,621/5,030 Matched: 1,621/1,621 | 597/1,526 605/1,482 | 0.791 (0.706-0.885) | <0.0001 | 0.039 | 0.647 |
| Age: 65-74 years | Query: 3,203/8,725 Pre-match: 2,867/7,928 Matched: 2,867/2,867 | 1,135/2,653 1,213/2,585 | 0.763 (0.704-0.828) | <0.0001 | 0.051 |  |
| Age: >=75 years | Query: 3,526/10,100 Pre-match: 3,004/8,759 Matched: 3,004/3,004 | 1,350/2,765 1,368/2,688 | 0.804 (0.745-0.867) | <0.0001 | 0.038 |  |
| ICI class: PD-1 only | Query: 6,683/19,145 Pre-match: 6,014/17,421 Matched: 6,014/6,014 | 2,456/5,591 2,582/5,440 | 0.760 (0.719-0.803) | <0.0001 | 0.045 | 0.204 |
| ICI class: PD-L1 only | Query: 1,298/4,431 Pre-match: 1,076/3,743 Matched: 1,075/1,075 | 476/979 485/947 | 0.809 (0.713-0.918) | 0.0010 | 0.063 |  |
| ICI class: CTLA-4-containing | Query: 608/1,378 Pre-match: 497/1,208 Matched: 489/489 | 194/445 192/440 | 0.903 (0.740-1.103) | 0.3181 | 0.074 |  |
| Metastatic disease: Yes | Query: 3,275/7,948 Pre-match: 2,859/6,933 Matched: 2,858/2,858 | 1,240/2,611 1,299/2,488 | 0.736 (0.681-0.796) | <0.0001 | 0.043 | 0.083 |
| Metastatic disease: No | Query: 5,256/15,261 Pre-match: 4,612/13,703 Matched: 4,612/4,612 | 1,832/4,316 1,847/4,246 | 0.805 (0.755-0.859) | <0.0001 | 0.071 |  |
| Baseline systemic steroids: Yes | Query: 4,927/13,903 Pre-match: 4,498/12,747 Matched: 4,498/4,498 | 1,863/4,126 1,870/3,991 | 0.793 (0.744-0.846) | <0.0001 | 0.035 | 0.255 |
| Baseline systemic steroids: No | Query: 3,604/10,420 Pre-match: 2,963/8,893 Matched: 2,960/2,960 | 1,216/2,788 1,348/2,751 | 0.748 (0.692-0.808) | <0.0001 | 0.047 |  |
| Concomitant chemotherapy: Yes | Query: 4,483/13,851 Pre-match: 3,982/12,450 Matched: 3,981/3,981 | 1,672/3,680 1,731/3,568 | 0.790 (0.739-0.845) | <0.0001 | 0.046 | 0.593 |
| Concomitant chemotherapy: No | Query: 4,048/10,472 Pre-match: 3,552/9,379 Matched: 3,552/3,552 | 1,433/3,301 1,504/3,241 | 0.769 (0.715-0.827) | <0.0001 | 0.053 |  |

For each independently reconstructed subgroup-specific cohort, the estimand was the post-landmark hazard ratio for all-cause mortality during days 181-1825 comparing patients with versus without an early endocrine diagnosis within that subgroup. The same prespecified 1:1 propensity-score matching framework and balance threshold (absolute SMD <0.10) were applied. No subgroup-specific analysis had a postmatching SMD >0.10. P for heterogeneity was calculated from subgroup-specific log hazard ratios and standard errors derived from their 95% confidence intervals; it is a summary-level between-stratum heterogeneity test rather than a patient-level Cox interaction term.
