# Supplementary material for: Early endocrine diagnoses and subsequent survival after immune checkpoint inhibitor initiation in non-small cell lung cancer: a 180-day landmark cohort study
Source: Front Oncol. 2026 Sep 17;16:1957383. doi: 10.3389/fonc.2026.1957383 (PMC13629181; doi:10.3389/fonc.2026.1957383)
Supplement: Supplementary file 6 [file Table6.docx]

**Supplementary Table S4. Operational definitions, analytic risk sets, and results for revised secondary outcomes.**

**Panel A. Outcome-specific analytic design and risk-set construction.**

| **Outcome** | **Source analysis** | **Prior-event handling** | **Analytic risk set and matching** | **Outcome window** |
| --- | --- | --- | --- | --- |
| Time to first recorded prespecified subsequent systemic therapy or death (TTNT-D) | Independent TTNT-D-specific analysis | Patients with any qualifying subsequent-therapy code from the beginning of the available record through day 180 after first ICI were excluded in the cohort definition before matching. No outcome-level prior-event exclusion was subsequently applied. | Query N: 2,114 vs 5,065; PSM-eligible N: 1,876 vs 4,670; matched N: 1,876 vs 1,876; maximum postmatch absolute SMD: 0.047 | Days 181-1825 |
| Severe infection | Primary matched landmark cohort | No outcome-level prior-event exclusion; patients with previous sepsis-related records were retained. | Query N: 8,531 vs 24,323; PSM-eligible N: 7,696 vs 22,273; matched N: 7,696 vs 7,696; maximum postmatch absolute SMD: 0.041 | Days 181-1825 |
| Healthcare utilization | Primary matched landmark cohort | No outcome-level prior-event exclusion; patients with previous healthcare encounters were retained. | Query N: 8,531 vs 24,323; PSM-eligible N: 7,696 vs 22,273; matched N: 7,696 vs 7,696; maximum postmatch absolute SMD: 0.041 | Days 181-1825 |

The original hospice/palliative-care endpoint was removed from the revised analysis because outcome-level prior-event exclusion was applied after matching, producing unequal analytic denominators without post-exclusion balance diagnostics, and because coded records could not reliably distinguish new hospice initiation from recurrent or administrative documentation.

**Panel B. Secondary-outcome results and competing-risk interpretation.**

| **Outcome** | **Matched N, exposed / control** | **Events/N, exposed / control** | **HR (95% CI)** | **Log-rank P** | **Death/competing-risk handling** |
| --- | --- | --- | --- | --- | --- |
| Time to first recorded prespecified subsequent systemic therapy or death (TTNT-D) | 1,876 / 1,876 | 866/1,876 843/1,876 | 0.849 (0.773-0.934) | 0.0007 | Death was included in the OR composite. |
| Severe infection | 7,696 / 7,696 | 1,265/7,696 866/7,696 | 1.255 (1.151-1.369) | <0.0001 | No formal competing-risk regression was available; interpreted as an exploratory post-landmark nonmortality endpoint. |
| Healthcare utilization | 7,696 / 7,696 | 4,984/7,696 4,306/7,696 | 1.055 (1.013-1.099) | 0.0101 | No formal competing-risk regression was available; interpreted as an exploratory post-landmark nonmortality endpoint. |

**Panel C. Full component code definitions.**

| **Outcome** | **Data domain** | **Coding system and code** | **Component description** |
| --- | --- | --- | --- |
| TTNT-D | Treatment concept | TriNetX GlobalOncology: TNX:TREATMENT:100312 | Chemotherapy, Line 2 (ATC) |
| TTNT-D | Medication | RxNorm: 337525 | Erlotinib |
| TTNT-D | Medication | RxNorm: 328134 | Gefitinib |
| TTNT-D | Medication | RxNorm: 1430438 | Afatinib |
| TTNT-D | Medication | RxNorm: 2058849 | Dacomitinib |
| TTNT-D | Medication | RxNorm: 1721560 | Osimertinib |
| TTNT-D | Medication | RxNorm: 2549199 | Amivantamab |
| TTNT-D | Procedure | HCPCS: J9061 | Injection, amivantamab-vmjw, 2 mg |
| TTNT-D | Medication | RxNorm: 2555 | Cisplatin |
| TTNT-D | Procedure | HCPCS: J9060 | Injection, cisplatin, powder or solution, 10 mg |
| TTNT-D | Medication | RxNorm: 40048 | Carboplatin |
| TTNT-D | Procedure | HCPCS: J9045 | Injection, carboplatin, 50 mg |
| TTNT-D | Medication | RxNorm: 68446 | Pemetrexed |
| TTNT-D | Medication | RxNorm: 56946 | Paclitaxel |
| TTNT-D | Procedure | HCPCS: J9267 | Injection, paclitaxel, 1 mg |
| TTNT-D | Procedure | HCPCS: J9264 | Injection, paclitaxel protein-bound particles, 1 mg |
| TTNT-D | Medication | RxNorm: 72962 | Docetaxel |
| TTNT-D | Procedure | HCPCS: J9171 | Injection, docetaxel, 1 mg |
| TTNT-D | Medication | RxNorm: 12574 | Gemcitabine |
| TTNT-D | Procedure | HCPCS: J9201 | Injection, gemcitabine hydrochloride, not otherwise specified, 200 mg |
| TTNT-D | Medication | RxNorm: 39541 | Vinorelbine |
| TTNT-D | Medication | RxNorm: 4179 | Etoposide |
| TTNT-D | Procedure | HCPCS: J9181 | Injection, etoposide, 10 mg |
| TTNT-D | Diagnosis | ICD-10-CM: R99 | Ill-defined and unknown cause of mortality |
| TTNT-D | Demographic status | TriNetX: Deceased | Deceased |
| TTNT-D | Diagnosis | ICD-10-CM: I46 | Cardiac arrest |
| TTNT-D | Diagnosis | ICD-10-CM: R09.2 | Respiratory arrest |
| Severe infection | Diagnosis | ICD-10-CM: A41.9 | Sepsis, unspecified organism |
| Severe infection | Diagnosis | ICD-10-CM: A41 | Other sepsis |
| Severe infection | Diagnosis | ICD-10-CM: R65.21 | Severe sepsis with septic shock |
| Healthcare utilization | Visit | HL7 v3 visit type: EMER | Emergency visit |
| Healthcare utilization | Visit | HL7 v3 visit type: ACUTE | Inpatient acute visit |
| Healthcare utilization | Visit | HL7 v3 visit type: IMP | Inpatient encounter |
| Healthcare utilization | Visit | HL7 v3 visit type: NONAC | Inpatient non-acute visit |
| Healthcare utilization | Visit | HL7 v3 visit type: SS | Short-stay visit |

All multi-component outcomes were implemented using OR logic. TTNT-D is an operational EHR endpoint and does not constitute radiographically adjudicated progression or a formally reconstructed treatment line. Abbreviations: HR, hazard ratio; PSM, propensity-score matching; SMD, standardized mean difference.
